# Graphene Oxide Protected Copper Benzene-1,3,5-Tricarboxylate for Clean Energy Gas Adsorption

**DOI:** 10.3390/nano10061182

**Published:** 2020-06-17

**Authors:** Andrea Domán, Szilvia Klébert, János Madarász, György Sáfrán, Ying Wang, Krisztina László

**Affiliations:** 1 Department of Physical Chemistry and Materials Science, Budapest University of Technology and Economics, Budafoki út 8., H-1521 Budapest, Hungary; doman.andrea@mail.bme.hu; 2 Institute of Materials and Environmental Chemistry, Research Centre for Natural Sciences, Eötvös Loránd Research Network, Magyar tudósok körútja 2., H-1117 Budapest, Hungary; klebert.szilvia@ttk.hu; 3 Department of Inorganic and Analytical Chemistry, Budapest University of Technology and Economics, Szt. Gellért tér 4., H-1521 Budapest, Hungary; madarasz@mail.bme.hu; 4 Research Institute for Technical Physics and Materials Science, Eötvös Loránd Research Network, Konkoly Thege M. út 29-33., H-1121 Budapest, Hungary; safran.gyorgy@energia.mta.hu; 5 College of Environmental Science and Engineering, Tongji University, 1239 Siping Road, Shanghai 200092, China; yingwang@tongji.edu.cn

**Keywords:** MOF, HKUST-1, GO, composite, adsorption gas storage, water vapor

## Abstract

Among microporous storage materials copper benzene-1,3,5-tricarboxylate (CuBTC MOF, Cu_3_(BTC)_2_ or HKUST-1) holds the greatest potential for clean energy gases. However, its usefulness is challenged by water vapor, either in the gas to be stored or in the environment. To determine the protection potential of graphene oxide (GO) HKUST-1@GO composites containing 0–25% GO were synthesized and studied. In the highest concentration, GO was found to strongly affect HKUST-1 crystal growth in solvothermal conditions by increasing the pH of the reaction mixture. Otherwise, the GO content had practically no influence on the H_2_, CH_4_ and CO_2_ storage capacities, which were very similar to those from the findings of other groups. The water vapor resistance of a selected composite was compared to that of HKUST-1. Powder X-ray diffraction (XRD), scanning electron microscopy (SEM), transmission electron microscopy (TEM), thermogravimetric (TG/DTG) and N_2_ adsorption techniques were used to monitor the changes in the crystal and pore structure. It was found that GO saves the copper–carboxyl coordination bonds by sacrificing the ester groups, formed during the solvothermal synthesis, between ethanol and the carboxyl groups on the GO sheets.

## 1. Introduction

Since human activity has an increasingly damaging effect on the environment, the reduction of air pollution and the development of green technologies are now crucial. The rising levels of carbon dioxide in the atmosphere has led to the worldwide problem of climate change. As the transport sector, which contributes significantly to energy consumption at a global level, relies mainly on fossil fuels, it is one of the main sources of carbon dioxide emission [1]. The separation and capture of carbon dioxide would help to alleviate the greenhouse effect. Additionally, the use of clean energy gases as alternative fuels like natural gas or hydrogen could reduce carbon dioxide emission significantly. However, no economically viable solution has yet been found for storage of these gases. Nonetheless, adsorption gas storage seems to be a promising technology for this purpose, but its use requires the development of suitable adsorbents [2].

Metal-organic-frameworks (MOFs) are a recognized class of porous materials with outstandingly large adsorption capacity, which makes them excellent candidates for adsorption-based applications [3,4]. MOFs are composed of multivalent metal ions or clusters linked together by organic ligands, thereby creating an open framework with an ordered and permanent pore structure [5,6]. Copper benzene-1,3,5-tricarboxilate (Cu_3_BTC_2_ or HKUST-1 [7]) is one of the most widely researched MOFs. In its paddle-wheel secondary building unit (SBU), four carboxylate groups of four BTC^3−^ ligands link together two copper ions. In the open framework, each copper (II) cation has an unsaturated binding site. During the synthesis, these unsaturated binding sites and the pore structure are occupied by solvent molecules. The release of the adsorbed solvent molecules from the entire pore volume, so-called activation, is required to achieve maximum adsorption capacity, which is essential in gas storage [8]. However, this activation significantly reduces the stability of the MOF in the presence of water vapor and challenges its application [9].

Nevertheless, HKUST-1 has outstanding potential in gas storage. As an efficient CO_2_ adsorbent, this MOF can adsorb CO_2_ from 2.5 to 4.2 mmol·g^−1^ under atmospheric conditions [10,11]. In terms of methane and hydrogen adsorption, it is still one of the most promising adsorbents among several MOFs [12,13]. Its methane adsorption performance is close to the volumetric US DoE (Department of Energy) target (263 cm^3^ CH_4_/cm^3^ MOF at 35 bar) established for economical natural gas storage [14,15]. Theoretically, HKUST-1 is also the best paddle-wheel-based structure for hydrogen adsorption, but in itself, its performance is far from the commercial target [1,16].

The efficiency, in terms of gas storage, is determined by several properties. While the most important factors are probably a suitable pore structure and high adsorption capacity, fast adsorption/desorption kinetics, reversibility of storage, packing density, mechanical and chemical stability, inertness and thermal conductivity are also influential [17]. The ability to release and take up heat is a substantial issue, as the heat generated during adsorption can diminish the gas adsorption capacity, and endothermic desorption reduces the retrievable amount [18].

An ideal association of materials to form composite systems may offer a solution to overcome the limitations of MOFs, such as water sensitivity or mechanical weakness [19,20,21]. Graphene and graphene derivatives are excellent candidates for associates, since it is proved that in hybrid systems, they can improve water resistance, as well as electrical, thermal and mechanical properties [22,23]. Graphene, ideally, is a single sheet of graphite with a honeycomb-like lattice structure. An outstanding thermal conductivity (3000–5000 W·m^−1^·K^−1^) and high theoretical specific surface area (2630 m^2^·g^−1^) are among its remarkable properties. Graphene oxide (GO), often produced by the exfoliation of graphite [24], has attracted substantial interest as an intermediate for graphene manufacturing. A further reduction step leads to the formation of reduced graphene oxide (rGO) [25]. The chemical and structural defects present—albeit to a different extent—in these graphene derivatives degrade some of the favorable properties of graphene, including electrical and thermal conductivity. By contrast, their functional groups make GO and rGO advantageous compared to pristine graphene, in terms of chemical functionalization or tuneable hydrophilic, electrical and optical properties [26].

Although a wide variety of MOFs—e.g., MOF-5 [27], ZIF-8 [28], UiO-66 [29], MIL-125 [30], Cr-MIL-53 [31], MIL-101 [32], MOF-505 [33], Al-MOF-520 [34], Zr-MOF [35], Ni-MOF [36] and NiDOBDC [37]—have been used in MOF@GO composites and tested for gas adsorption, none of these groups investigated all of the three gases CH_4_, H_2_ and CO_2_ on the same system. In this paper, we focus on HKUST-1-based systems.

The combination of the unique features of HKUST-1 and graphene or graphene derivatives was found to improve the performance of the associated system in several applications. Recent review articles on the subject confirm the interest in such composites [38,39,40]. The enhancement of the electrochemical catalytic activity of HKUST-1@graphene derivative composite materials enabled the development of electrochemical sensors for H_2_O_2_ [41], NH_3_ [42], paracetamol and dopamine [43]. The HKUST-1@GO composite has been found to be an efficient ibuprofen delivery system [44]. HKUST-1@GO composites obtained by Li et al. through room-temperature solvent-free mechanochemical synthesis improved the adsorption properties of HKUST-1 for toluene vapor [45]. In that work, the optimum GO content in the composite proved to be 5%. Besides, they observed increased water stability after 2–10 h direct contact with liquid water. The excellent electrical characteristics of the composites also make them promising supercapacitor electrode materials [46,47]. MOF-GO composites have excelled in gas adsorption applications as well [48,49,50,51]. The potential of MOF@GO, particularly HKUST-1@GO composites, in gas adsorption—including that of CH_4_, CO_2_ and H_2_—has been widely studied [52,53,54,55,56,57,58,59,60,61,62,63,64,65,66]. Huang et al. prepared HKUST-1@GO composite powders with 0.5–5 wt.% GO content for the separation of CO_2_/CH_4_. Upon the introduction of 1 wt.% GO, the Brunauer–Emmett–Teller (BET) surface area and the pore volume increased by more than 20% with respect to the parent HKUST-1. Furthermore, it exhibited an almost 30% higher CO_2_ adsorption capacity, up to 8.19 mmol·g^−1^ at 1 bar and 273 K, whereas the CH_4_ adsorption capacity was nearly unchanged. A dual-site Langmuir–Freundlich (DSLF) model was applied to fit the experimental isotherm data for CH_4_ and CO_2_ and the ideal adsorbed solution theory (IAST) to predict the selectivity of the samples toward CO_2_ vs. CH_4_ for equimolar CO_2_/CH_4_ mixtures. The predicted isotherms showed that at 1 bar, the CO_2_/CH_4_ adsorption selectivity of HKUST-1@GO increased to 14, almost twice that of HKUST-1 [52]. Zhao et al. also used HKUST-1@GO powder with 10% GO content for CO_2_ capture. Although the apparent surface area and pore volume decreased slightly due to the GO, the CO_2_ adsorption capacity obtained was enhanced by 38% with respect to that of the parent MOF. The CO_2_/N_2_ selectivity indicated that the composite is efficient for CO_2_ separation as well [53]. Al-Naddaf et al. investigated the CH_4_ adsorption performance of HKUST-1@graphene derivatives. They used GO, rGO and carboxyl-functionalized GO (fGO). All three types of nanocomposite powders exhibited higher surface areas and porosity than the pristine MOF. Their HKUST-1@rGO nanocomposite with 10 wt.% rGO displayed the best performance, with approximately 30% higher methane delivery capacity in the pressure range 5.8–65 bar at room temperature compared to pristine HKUST-1 [54]. Sun et al. prepared GO- and rGO-containing associated systems with HKUST-1. The presence of rGO reduced the apparent surface area owing to the agglomeration of rGO particles but improved the thermal stability. They also found that the crystallinity of HKUST-1 in the associated material was maintained after being exposed to 55% relative humidity for one month [67]. The amount of added carbon nanoparticle (typically 10 wt.%) and the synthesis route selected substantially influenced the final pore structure and apparent surface area. The gas adsorption properties of the composites were very often inferior to those of the pristine HKUST-1 [41,42,43,46,68]. Nevertheless, a low GO content (1–10%) preserved the adsorption properties [53] or even improved them [44,45,54]. While the influence of the humidity on the gas adsorption properties of MOFs has been recognized by several groups, systematic knowledge on the subject is still limited [45,67,69].

This work aims to reveal the effect of GO on the atmospheric adsorption ability of HKUST-1@GO for “clean energy gases” (CH_4_ and H_2_) and CO_2_ under static conditions. Furthermore, the presence of GO as a potential protective agent in humid atmosphere is investigated. HKUST-1@GO composites with 9–24% GO content were obtained by the solvothermal reaction of the MOF precursors in the presence of GO. The GO was used as a suspension, obtained directly from the Hummers process in order to avoid the drying and re-suspension of the GO. The GO content was increased systematically in the precursor solution from 1 to 2.5 g·L^−1^. GO is expected to improve the water stability of composite systems with respect to HKUST-1 while maintaining, or even improving, the excellent gas adsorption properties of HKUST-1.

## 2. Materials and Methods

### 2.1. Materials

Benzene-1,3,5-tricarboxylic acid (H_3_BTC), copper(II) nitrate trihydrate (Cu(NO_3_)_2_·3H_2_O) (99.5%) and ethanol (CH_3_CH_2_OH) (99.5%), purchased from Merck (Budapest, Hungary), were used without further purification. For the preparation of the graphene oxide (GO) suspension, natural graphite (Graphite Týn, Týn nad Vltavou, Czech Republic) was used as a precursor; sulphuric acid (H_2_SO_4_) (95–97%), potassium permanganate (KMnO_4_), hydrogen peroxide (H_2_O_2_) and hydrochloric acid (HCl) (37%) were obtained from Merck (Budapest, Hungary); and phosphoric acid (H_3_PO_4_) (85%) was obtained from Reanal (Budapest, Hungary). High purity water of Millipore grade was used for the synthesis.

### 2.2. Sample Preparation

HKUST-1 (C_18_H_6_Cu_3_O_12_, Mw 604.87 g/mol) was synthesized under solvothermal conditions according to Wang et al. [70]. Briefly, 10 mL of 83.3 mM H_3_BTC dissolved in ethanol was thoroughly mixed with a stoichiometric amount of the aqueous solution Cu(NO_3_)_2_·3H_2_O (125 mmol·L^−1^, 10 mL). Air was eliminated from the mixture by bubbling with argon prior to sealing the autoclave. The mixture was kept at 80 °C for 24 h. The turquoise crystals obtained were filtered and washed with ethanol and dried in air.

The improved Hummers’ method was used to prepare the GO [71]. The pristine GO suspension was purified by successive centrifugation (Jouan BR4i Multifunction Centrifuge, Thermo Scientific, Waltham, MA, USA; 5000 min^−1^) and thorough washing with 1 mol·L^−1^ HCl and doubly distilled water [72]. The suspension contained 1.1 w/w% GO with a p*K*a close to 5 [73]. The GO was used in this suspended form. From the Raman analysis of the suspension, the intensity ratio of the G and D bands I_G_/I_D_ = 1. The apparent surface area of the freeze-dried GO was 21 m^2^·g^−1^. The C/O atomic ratio from the XPS measurement was 2.6 [74]. Further characteristics are reported in [72,75].

The composite samples were prepared as pristine HKUST-1, in the presence of 1, 1.5, 2 and 2.5 g·L^−1^ GO. The composites are labelled as HKUST-1@GO-c_GO_, where c_GO_ is the GO concentration in the reaction mixture. HKUST-1@GO-1 thus refers to a HKUST-1@GO composite material where the GO concentration in the reaction mixture was 1 g·L^−1^. Air-dried samples were homogenized in a mortar before further measurements.

### 2.3. Sample Characterization

Scanning electron microscopy (SEM, JEOL JSM 6380LA, Jeol Ltd., Tokyo, Japan) and conventional and high resolution transmission electron microscopy (200 kV Philips CM20 TEM, Philips, Amsterdam, The Netherlands and 300 kV JEOL 3010 HRTEM, Jeol Ltd., Tokyo, Japan) were used to characterize the morphology, grain and grain size distribution of the samples. In the case of SEM, the samples were fixed to copper sample holders with conductive carbon adhesive tapes. Gold coating (JEOL JFC-1200, Jeol Ltd., Tokyo, Japan) was applied to increase the conductivity of the samples. For TEM and HRTEM imaging, the samples were drop-dried on carbon-coated microgrids. To measure the crystal sizes on the SEM images, the JMicroVision 1.2.7 program (https://jmicrovision.github.io) was used. The typical crystal size was estimated from the size of about 100 crystals in each sample.

Powder X-ray diffraction (XRD) patterns were obtained in the range 2θ = 4°–84° with an X’pert Pro MPD (PANalytical Bv., Almelo, The Netherlands) X-ray diffractometer using an X’celerator type detector and Cu K_α_ radiation with a Ni filter foil (λ = 1.5408 Å) and a “zero-background Si single crystal” sample holder. Phase identification was assisted by the Search&Match algorithm of the HighScore Plus (PANalyticalBv., Almelo, The Netherlands) software, based on either the international Powder Diffraction File (PDF4+, Release 2015, International Centre of Diffraction Data, ICDD, Pennsylvania, USA), or The Cambridge Structural Database (CSD-Enterprise, version 5.37, Cambridge Crystallographic Data Centre, CCDC [76]) using the built-in powder pattern generator algorithm of the Mercury program [77].

Nitrogen adsorption–desorption isotherms were measured at −196 °C by a NOVA 2000e (Quantachrome, Boynton Beach, FL, USA) volumetric computer-controlled surface analyzer. The apparent surface area *S*_BET_ was calculated using the Brunauer–Emmett–Teller (BET) model [78]. The total pore volume *V*_tot_ was derived from the amount of N_2_ adsorbed at relative pressure *p*/*p*_0_ → 1, assuming that the pores were filled with liquid adsorbate. The micropore volume *V*_micro_ was obtained from the Dubinin–Radushkevich (DR) plot [79]. Pore size distributions were calculated by the Barrett–Joyner–Halenda (BJH) method [80]. Transformation of the primary adsorption data was performed with the Quantachrome ASiQwin software (version 3.0, Quantachrome, Boynton Beach, FL, USA). All the samples, also including those exposed to water vapor, were outgassed in vacuum at 110 °C (activation) prior to the nitrogen adsorption measurements.

### 2.4. Hydrogen, Methane and Carbon Dioxide Adsorption

Methane and CO_2_ adsorption–desorption isotherms were measured at 0 °C with an AUTOSORB-1 (Quantachrome, Boynton Beach, FL, USA) computer-controlled analyser. An Autosorb 1C (Quantachrome, Boynton Beach, FL, USA) static volumetric instrument was used to perform hydrogen sorption experiments with high purity hydrogen (99.999%) at −196 °C. All the samples were outgassed in vacuum at 110 °C (activation) prior to the gas adsorption measurements.

### 2.5. Effect of Humidity

Air-dried (20 °C for 48 h) and activated (110 °C, 24 h evacuation) samples were exposed to humid atmospheres (relative humidity RH = 11% and 85%) at 25 °C for 21 days. The samples were designated by name, drying temperature and relative humidity. HKUST-1_110_11 thus refers to a HKUST-1 sample evacuated at 110 °C in vacuum before exposure to RH 11%. A simultaneous TG/DTA instrument (STD 2960 Simultaneous DTA-TGA, TA Instruments Inc., New Castle, Delaware, DE, USA) was employed for the thermal analysis of these samples (open Pt crucible; heating rate, 10 °C min^−1^; dry air flow, 130 cm^3^·min^−1^). The cured samples were analyzed immediately with no further treatment except the nitrogen adsorption measurements.

## 3. Results and Discussion

### 3.1. Characterization of the Composite Materials

Five samples of different GO content were synthesized by systematically increasing the GO concentration (1, 1.5, 2, 2.5 g·L^−1^) in the precursor mixture (Table 1). Unlike the synthesis routes reported by other groups [41,42,44,52,53,54,58,61,69], here, GO was used in suspended form as obtained from the improved Hummers’ method [71]. In this way, the drying and re-suspension of the GO can be circumvented.

**Figure 1:**
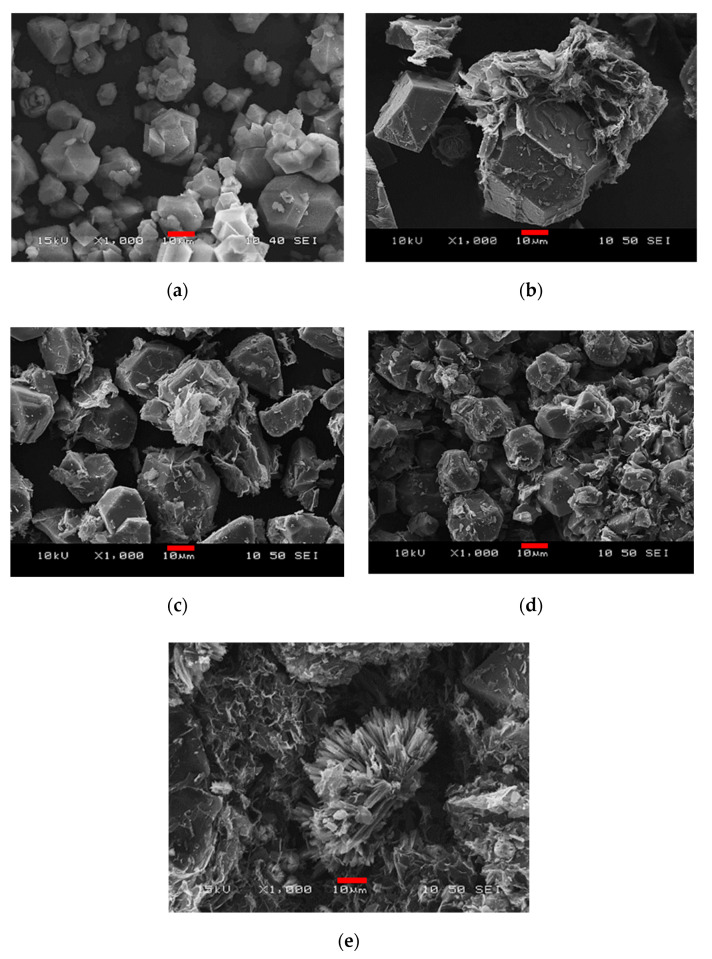
Scanning electron microscopy (SEM) images of (**a**) copper benzene-1,3,5-tricarboxylate (Cu_3_(BTC)_2_ or HKUST-1), (**b**) HKUST-1@GO (graphene oxide)-1, (**c**) HKUST-1@GO-1.5, (**d**) HKUST-1@GO-2 and (**e**) HKUST-1@GO-2.5. The scalebar is 10 µm.

The presence of GO hardly influences the yield of MOF except when 50 mg of GO is added; here, the yield drops by almost 25%. Figure 1 compares the typical SEM images of the samples. The effect of GO on the HKUST-1 crystals is spectacular. The addition of 20 mg of GO practically doubles the size of some of the crystals (Figure 1b). A further increase in the GO concentration (Figure 1c,d) gradually decreases the crystals to the typical size of “free” HKUST-1 (Figure 1a). At the highest GO concentration tested, rod- and flower-like structures (Figure 1e) appear in addition to the usual polyhedral crystals. No such formations were observed in the other samples. The GO sheets adhere strongly to the MOF but wrap around the HKUST-1 crystals only partially, independently of the amount added.

We assume that the large amount of GO sheets acts as a physical barrier, inhibiting or slowing down the crystal growth and thus leading to the rod- and flower-like structures and a reduced yield (Figure 1e). Moreover, the addition of the GO suspension, owing to the acid/base properties of the GO itself, necessarily influences the self-assembly of the HKUST-1 precursors and may also contribute to this spectacular morphology. Our spectrophotometric and zeta potential measurements confirmed that the p*K*a of this GO is in the region of 4–5 [73].

**Figure 2:**
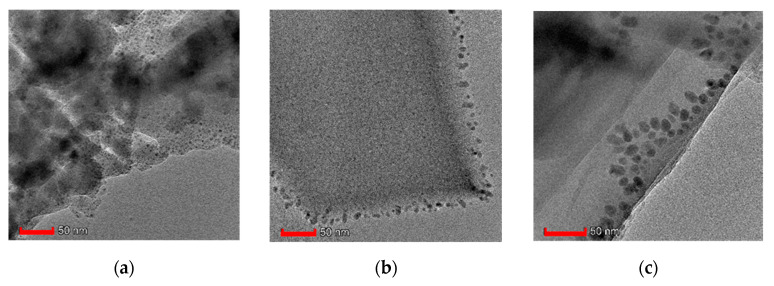
High resolution transmission electron microscopy (HRTEM) images of (**a**) HKUST-1, (**b**) HKUST-1@GO-1 and (**c**) HKUST-1@GO-2.5. The scalebar is 50 nm.

The HRTEM images in Figure 2 show that the bulky crystals of HKUST-1 are built up from nanosized crystalline particles that eventually ripen into larger crystals through oriented attachment [81]. As shown in our HRTEM, isolating HKUST and HKUST-GO from the reaction medium halts the ripening process, leaving some residual nanosized particles. In the pristine HKUST-1, the average size of the nanocrystals is ca 4.6 nm, while in HKUST-1@GO-1 and HKUST-1@GO-2.5, they are typically larger—around 7.6 and 11.0 nm, respectively. The larger nanocrystal size implies that the nucleation of HKUST-1 crystals and their oriented ripening may be slightly inhibited in the presence of GO.

**Figure 3:**
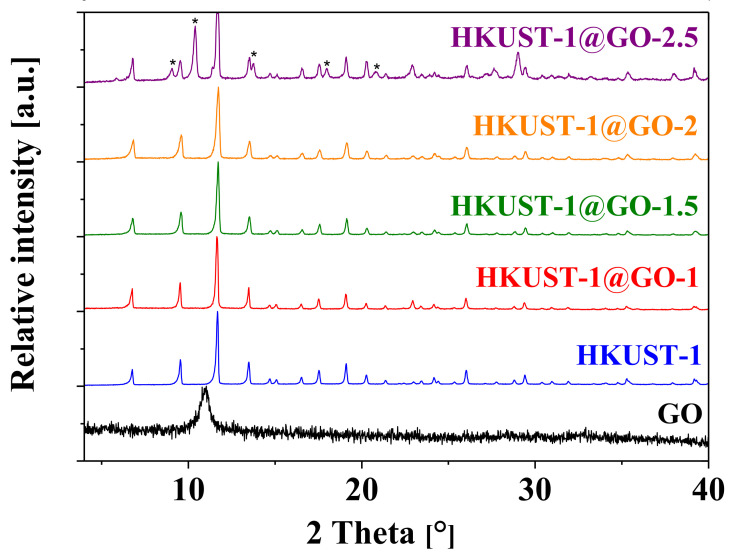
X-ray diffraction (XRD) patterns of the pristine GO, HKUST-1 and the composite materials with different GO contents. The peaks were normalized to the maximum intensity peak and shifted for better visibility. * marks unidentified peaks not belonging to HKUST-1.

Powder X-ray diffraction was used to identify the crystalline phase. The characteristic XRD pattern of HKUST-1 (PDF_00-062-1183, PDF4+ (Release 2020) database, http://www.icdd.com/) was clearly observed in all the composite systems (Figure 3.). In the diffractogram of the pure GO, the broadened peak centered at 2θ = 10.9° corresponds to a modus of spacing distribution 8.1 Å, deduced from the Bragg equation. This is slightly larger than the 9.1–9.5 Å found by other authors [58,63]. During the hydrothermal synthesis, GO undergoes further exfoliation [58,60,63,64]. High resolution images reveal that the GO sheets are well dispersed in the associated material and are directly attached to the HKUST-1 crystals. The diffraction results show no difference in the crystalline patterns except in the case of HKUST-1@GO-2.5. The latter diffractogram contains additional peaks, which may correspond to the different unusual structures.

Wang et al. used different modulators (sodium formate, sodium acetate and triethylamine) to control the morphology and size of HKUST-1 [70]. They demonstrated that pH can indeed significantly influence crystal growth by affecting the protonation of benzene-1,3,5-tricarboxylate (BTC) ligands and, thus, the crystal nucleation. Only rod-like structures were observed in the corresponding SEM images. Interestingly, the peak positions in the XRD patterns of crystals prepared in the presence of sodium formate (six equivalents with respect to BTC) are in good agreement with the additional peaks in the HKUST-1@GO-2.5 sample. Presumably, the effect of GO and sodium formate in the reaction mixture is very similar: they shift the original pH = 2.05 of the synthesis mixture to higher, but still acidic, values.

**Figure 4:**
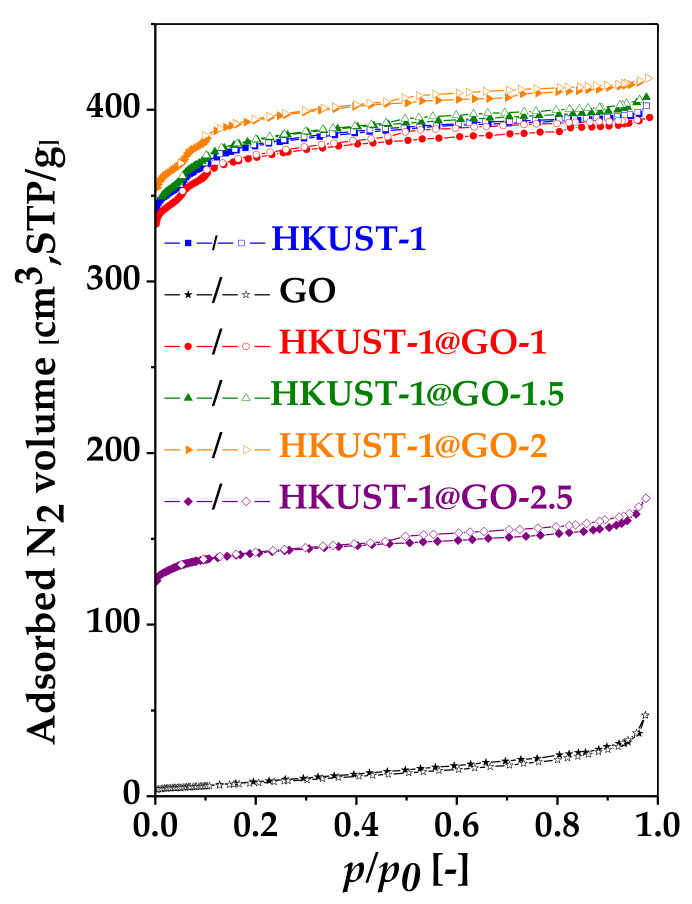
N_2_ adsorption–desorption isotherms (−196 °C) of the pristine GO, HKUST-1 and the composite materials with different GO contents. The adsorption and desorption branches are denoted by full and empty symbols, respectively.

Nitrogen adsorption–desorption isotherms were used to reveal the effect of GO on the porous texture (Figure 4 and Figure S1). The shape of the MOF-related isotherms, typical of microporous systems, is of Type Ib according to the recent IUPAC classification [82]. Unlike HKUST-1 itself, all the GO-containing samples exhibit a flat, elongated hysteresis loop of Type H4, often found with aggregated crystals. GO alone, without a deliberately designed multidimensional structure, is not a highly porous material. However, when it is dispersed in the composite systems, it adheres to the crystals, thus affecting the development of the pore structure. Only the 50 mg GO content in HKUST-1@GO-2.5 results in a significant deviation from the isotherm of pure HKUST-1. Therefore, it is not surprising that the values of *S*_BET_, *V*_micro_ and *V*_tot_ of the other composite systems are very similar and close to those of pure MOF (Table 1). The largest GO content produced a dramatic change: the values of *S*_BET_, *V*_micro_ and *V*_tot_ all decreased by about 60%. This may be related to the different crystal formations having different pore structures and thus limited adsorption properties. Although the mesopore volumes of the various samples are practically identical, the evolution of the hysteresis loops in the GO-containing samples is a sign of alteration of the texture in the mesopore range, even if these changes are beyond the sensitivity of the XRD technique. As no kernel files necessary for DFT based calculations are available for associated systems, the pore size distribution (PSD) was calculated with the BJH method (Figure S2). This method is limited to the mesopore range. The isotherms and these PSD curves concomitantly show the strong influence of GO in the micropore region.

**Figure 5:**
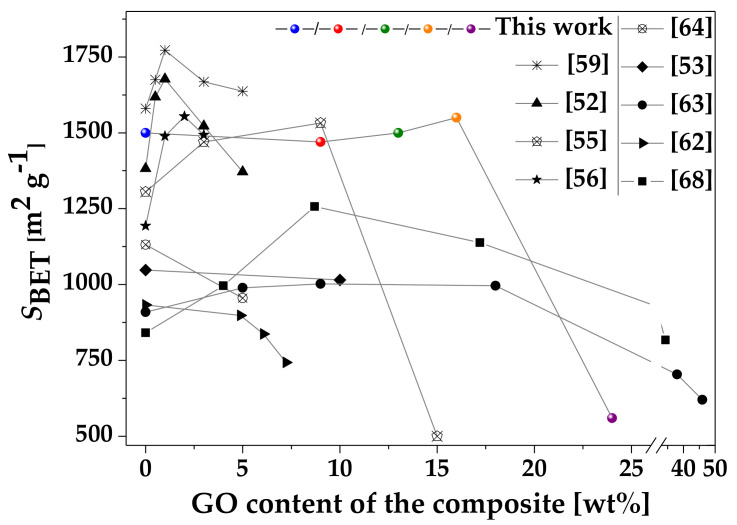
Influence of GO content on the apparent surface area of the associated HKUST-1@GO. Comparison of the present results and reference data.

The data extracted from nitrogen adsorption measurements (Table 1) show that the surface area of the associated samples is not a linear combination of the components. Figure 5 summarizes how the GO affects the apparent surface area of the composites. At lower GO, content there is synergism, but a higher GO content reduces the surface area. Even in the case of “pure” HKUST-1, the particle size–surface area relation is contradictory [70,83]. A comparative evaluation of the already published data is almost impossible as GO is a poorly defined material; its properties (size, surface chemistry, etc.) depend strongly on several factors including the synthesis conditions. Moreover, the formation of the associated material is also a complex process. The following elements of the mechanism have been recognized: (i) the oxygen-containing functional groups of the GO interact with the Cu^2+^ ions, and the GO sheets thus provide nucleation sites for HKUST-1 growth; (ii) the presence of GO reduces the spatial freedom for crystal growth; (iii) too much GO disturbs the crystal formation; (iv) at high GO concentration, GO can agglomerate, which also may lead to distorted structures [55,56,68]. The interactions between the oxygen-containing functional groups and the copper ions may result in new micro-, meso- and/or macro-pore formation [54,56,59,68]. Interestingly, none of these studies address the possible influence of GO on the pH of the synthesis medium [70]. To elucidate the surface area–particle size correlation and find a relevant interpretation in associated materials would require systematic experimental work dedicated to this question, particularly in GO-containing systems. This was not the aim of the present paper. Nevertheless, Figure 5 reveals that the apparent surface area of our samples is comparable to the highest values reported by other groups [52,53,54,55,56,58]. The concentration-dependent effect of GO implies that the GO content must be optimized according to the desired performance, e.g., to obtain the best adsorption properties.

### 3.2. Adsorption Capacity for Clean Energy Gases and CO_2_

**Figure 6:**
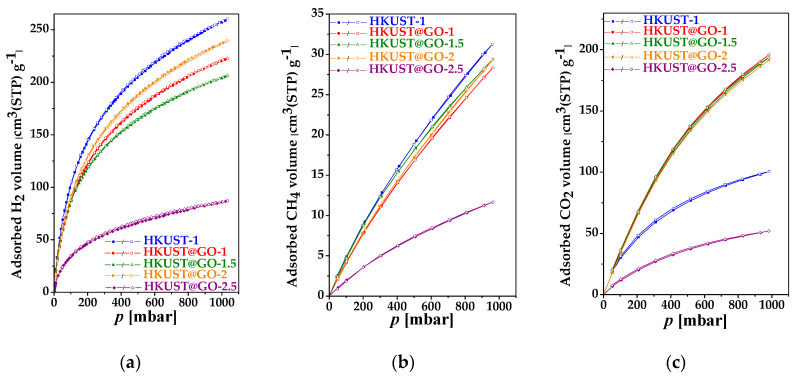
Adsorption capacity of the samples at atmospheric pressure for (**a**) H_2_ at −196 °C and (**b**) CH_4_ at 0 °C and (**c**) CO_2_ at 0 °C.

The atmospheric adsorption of the samples was tested for methane and carbon dioxide at 0 °C and for hydrogen at −196 °C (Figure 6, Table 2, reported as cm^3^·(STP)·g^−1^). The data in Table S1 (for comparison reported as mmol·g^−1^) show that the static adsorption capacity of our associated samples is comparable to that of the best-performing HKUST-1@GO composites reported by other groups. The aim of including CO_2_ in this study was two-fold. The nanoporous materials investigated in this work may be applicable to CO_2_ sequestration. On the other hand, CO_2_ is recommended by IUPAC as a routine test gas to reveal the pore structure in the ultra-microporous region, i.e., below 0.7 nm [82]. Figure 6c shows that in the range *p* → 0, all the CO_2_ isotherms (except HKUST-1@GO-2.5) overlap, which implies similar pore texture in the ultra-micropore range (Figure S3). The adsorption performance of N_2_, H_2_, CH_4_ and CO_2_ displays different trends according to GO content. A comparison of the sets of isotherms shows that only the HKUST-1@GO-2.5 sample again exhibits significant deviation from the other samples. The H_2_ adsorption capacity of the composite systems does not reach that of pristine HKUST-1 (11.5 mmol·g^−1^). HKUST-1@-GO-2 is the best, with 10.9 mmol·g^−1^.

The very similar CH_4_ adsorption capacities of all the GO-containing samples (except HKUST-1@GO-2.5), 1.3 ± 0.1 mmol·g^−1^ on average, is only slightly lower than that of pure HKUST-1. Although the CO_2_ adsorption capacity of HKUST-1 (4.0 mmol·g^−1^) is below the 6–8 mmol·g^−1^ reported by other groups [52,55,56,59], the uptake of the composites (except HKUST-1@GO-2.5), ca. 8.5 mmol·g^−1^, is very close to the values published by other authors [52,55,56,59]. On the basis of the morphological characteristics and the adsorption performance listed in Table 2, HKUST-1@GO-2 was selected for further investigation.

### 3.3. Effect of Water Vapor

Our previous work [9] revealed the destructive effect of water vapor on the structure of solvent-free HKUST-1. In order to study the influence of the GO as a potential protecting associate component under humid conditions, the behaviour of the pure HKUST-1 and the selected composite was investigated. HKUST-1 and HKUST-1@GO-2 samples dried at 20 °C under ambient conditions (40–50% RH) or activated at 110 °C (vacuum, 24 h) were exposed to atmospheres of RH 11% and 85% at 25 °C for 21 days. At ambient conditions, it is not possible to remove all the water molecules from the structure of HKUST-1, i.e., the pores remain partly occupied by water molecules. By contrast, activation at 110 °C removes all the water molecules, including those that decorate the “free” copper sites and are thus part of the crystal structure. The latter treatment leaves behind naked copper sites that are extremely vulnerable when re-exposed to water [9].

**Figure 7:**
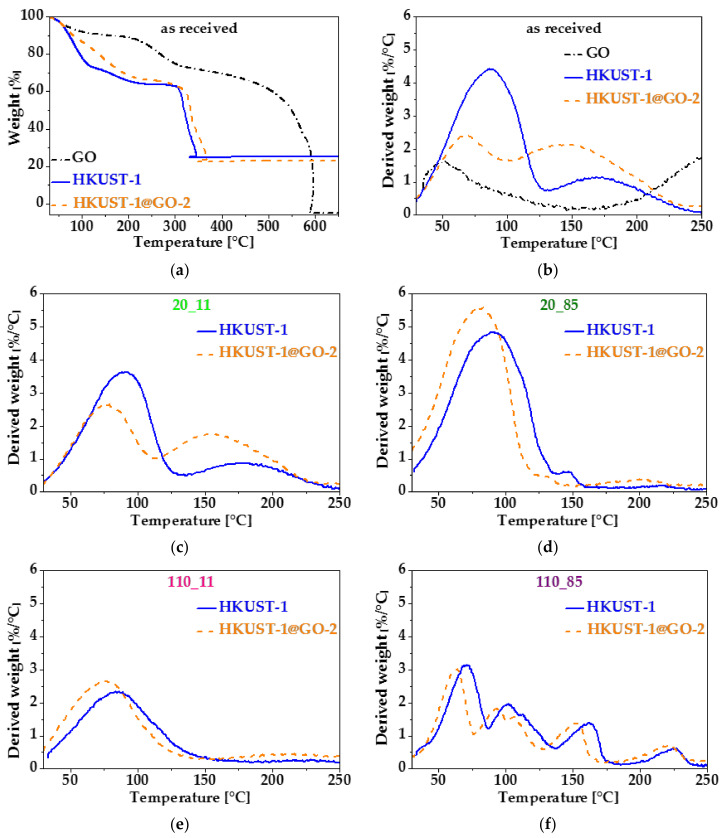
Comparison of the (**a**) weight loss (TG) and (**b**) corresponding derivative (weight loss rate, DTG) curves in the water loss region of parent and composite materials: GO (black, dash-dot), HKUST-1 (blue, solid), HKUST-1@-GO-2 (orange, dashed). DTG curves focusing on the water loss region of air-dried samples after exposure to (**c**) relative humidity (RH) 11% and (**d**) RH 85%. HKUST-1 and activated samples after exposure to (**e**) RH 11% and (**f**) RH 85%. Thermal analysis was carried out in air (130 cm^3^·min^−1^) at a heating rate of 10 °C·min^−1^.

Thermal analysis was performed on the as-received (20 °C, no vacuum) and cured (exposed to humidity) samples without further treatment (Figure 7 and Tables S3 and S4). Figure 7a shows the full thermogravimetric (TG) curves of HKUST-1@GO-2 and its individual components. In all cases, weight loss occurs in several steps. GO loses the physically adsorbed water prior to 150 °C, and above 200 °C, the oxygen-containing functional groups decompose. Around 550 °C, the GO starts to burn [75]. The thermogravimetric curves of HKUST-1 and HKUST-1@GO-2 are very similar.

The first step in the weight loss signal of HKUST-1 corresponds to physically bound water. The mass loss in the range 150–250 °C is related to the release of thermally hydrolysed ethanol from the ethyl ester of the H_3_BTC ligands formed during the solvothermal synthesis at the edges of the HKUST-1 crystals [84]. The sharp step at 300 °C marks the oxidation of the organic ligand. Figure 7b highlights the derivatives of the same curves up to 250 °C (a similar plot of all the as-received composites is shown in Figure S3). The full temperature scale TG curves and the slightly extended temperature range (up to 350 °C, Figure S4) of the latter derivative thermogravimetric (DTG) plots reveal that the “free” GO exhibits weight loss in the range 150−300 °C (here in air), due to decomposition of carboxylic and lactone groups [85]. Although during the solvothermal synthesis in the ethanol–water mixture, these groups may be converted to esters, which could have a higher temperature stability [60], the characteristic decay of GO around 550 °C disappears in all the composites. The anomalous shape of the HKUST-1-related response curves in the range 300–350 °C indicates the overheating of the sample by the hugely exothermic character of the oxidative decomposition of the organic spacers. (The curves were recorded as a function of sample temperature.) The heat generated and the presence of Cu as a potential catalyst result in the concurrent oxidation of the GO.

Figure 7b–f compares the weight loss rate (DTG) curves up to 250 °C of the corresponding HKUST-1 and composite samples having the same prehistory. The peak positions of composite materials are shifted to slightly lower temperatures, which may indicate that the release of the volatile species from the planar GO surface requires less energy than that from the narrow capillaries of the MOF. Figure 7b reveals that the ratio of the peaks from physisorbed water and chemisorbed ethanol are notably different in HKUST-1 and HKUST-1@GO-2, implying that the carboxyl groups of GO form ethyl esters during the solvothermal synthesis [60]. (The difference of the “water” peaks below 100 °C may come from the uncontrolled humidity of the laboratory air.) The similar shapes of the corresponding curves in Figure 7b,c reveal very similar thermal processes. RH 11% is below the RH of the condition before drying, i.e., the non-evacuated samples lose pore water, as reflected by the reduction in the water peak in both HKUST-1_20_11 and HKUST-1@GO-2_20_11. At RH 85%, both samples adsorb more water (Figure 7d). We may assume that the higher chemical potential of the water vapor results in the hydrolysis of the ester bonds. This is corroborated by the enhancement of the peak corresponding to the physisorbed solvents. The new peak that develops in the HKUST-1 sample at 150 °C is a sign of the decay of the HKUST-1 crystals exposed to the higher RH. In the composite sample, however, we assume that hydrolysis of the ester bonds thwarts the attack of the crystalline structure. Instead of eroding the copper–BTC coordinations within the pores, the water hydrolyses the easily accessible ester groups on the GO surface. The shift between the two curves in Figure 7d is a result of the higher ethanol content, i.e., higher volatility, released by the GO sites.

It is remarkable that, after removing all the water from the pores during the activation in the RH 11% medium, the amount of physisorbed solvent accommodated in the pores is much less than in any of the previous cases. As practically only one peak was observed in these samples, we assume that the available water molecules participate mainly in the ester hydrolysis (Figure 7e). The most spectacular change occurred when the activated samples were exposed to the higher RH atmosphere (Figure 7f). In the corresponding DTG curves, five peaks can be identified. It is reasonable to suppose that under these conditions, there is enough water to hydrolyse not only the ester bonds but also the Cu–carboxylic coordinations, leading to the desintegration of the HKUST-1 structure. Part of the water will coordinate with the copper sites and form strong coordination bonds with water. These are therefore released only at elevated temperatures, above 100 °C. As with HKUST_1, five different water binding sites were also recognised in the composite material [9]. The complete removal of the water during the activation leaves behind reactive Cu sites and easily accessible MOF porosity [9]. Therefore, the protecting effect of the GO ester groups cannot prevail.

**Figure 8:**
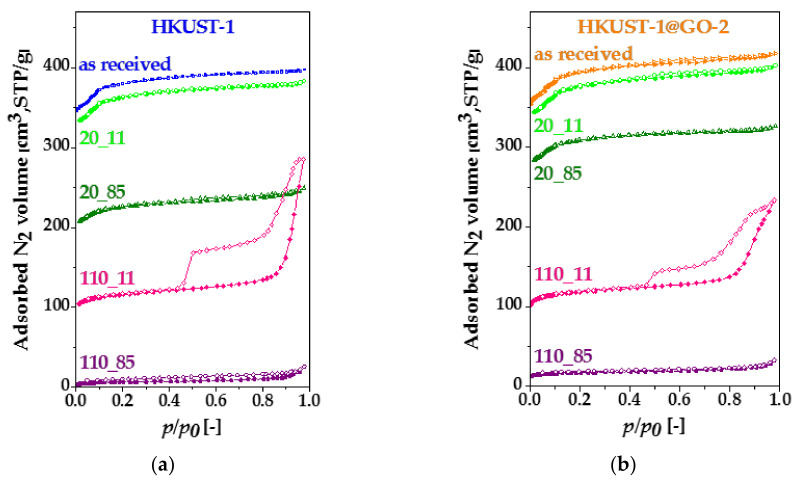
Nitrogen adsorption–desorption isotherms of (**a**) HKUST-1 and (**b**) HKUST-1@GO-2 before and after exposure to different relative humidities. Air-dried (20 °C, no vacuum) materials were exposed to RH 11% (20_11) and RH 85% (20_85); the activated (110 °C, vacuum) samples were exposed to RH 11% (110_11) and RH 85% (110_85). The adsorption and desorption branches are indicated by full and empty symbols, respectively.

The nitrogen adsorption/desorption isotherms also confirm the transformation of the pore structure (Figure 8). The changes in the isotherms of HKUST-1 (Figure 8a) and HKUST-1@GO-2 (Figure 8b) samples of identical prehistory show very similar trends. The data derived from the nitrogen adsorption measurements are listed in Table 3.

The isotherms with a different resolution and the pore size distribution of these systems are plotted in Figures S5 and S6, respectively. The exclusively microporous nature of the parent material was fully retained in the air-dried (20 °C, no vacuum) samples after exposure either to RH 11% or 85%. The isotherms in a more expanded scale and the pore size distributions are plotted in Figures S5, S7 and S8, respectively. The apparent surface area and the pore volumes of HKUST-1 and HKUST-1@GO-2 decreased only slightly after being exposed to RH 11%. The loss in the sorption capacities was more significant after RH 85%. Obviously, GO provides a remarkable degree of protection under this condition. Nevertheless, in spite of the more substantial loss of porosity, no changes in the shape of the isotherms were observed, i.e., only the microporous nature of the samples was maintained. The narrow hysteresis loop in Figures S5c and S7b could be an indicator of slight textural “erosion”. The effect of humid air was more radical when the water content of the samples was completely removed at 110 °C before exposure to the humid environment. In addition to the dramatic loss of adsorption capacity, the pore structure changed completely in both samples in RH 11%. This change is reflected in the transition of the isotherm from Type I to Type IVa. The pore volumes and the hysteresis loop of type H2 indicate the formation of mesopores. Hysteresis loops of Type H2 are typical in case of complex pore structures showing network effects [82].

**Figure 9:**
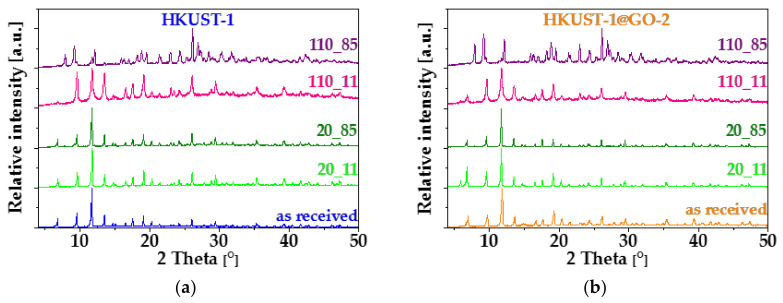
XRD profiles of (**a**) HKUST-1 and (**b**) HKUST-1@GO-2 before and after exposure to humid air. Blue and orange patterns belong to initial air-dried HKUST-1 and HKUST-1@GO-2, respectively. Initial air-dried (20 °C) and activated (110 °C, vacuum) materials were exposed to RH 11% and 85% at 25 °C for 21 days and then measured without further treatment.

Powder XRD was performed on the cured samples without further treatment. Figure 9 shows the XRD profiles of the HKUST-1 (Figure 9a) and of the composite (Figure 9b) samples with different prehistory. The same diffractograms of samples with identical prehistory are shown in Figure S9. The characteristic XRD pattern of HKUST-1 is clearly retained in all samples dried in air at 20 °C, independently of the conditions during the 3-week storage (samples with labels 20_11 and 20_85). In spite of the more substantial loss of porosity, no changes were observed in the corresponding XRD diffractograms.

**Figure 10:**
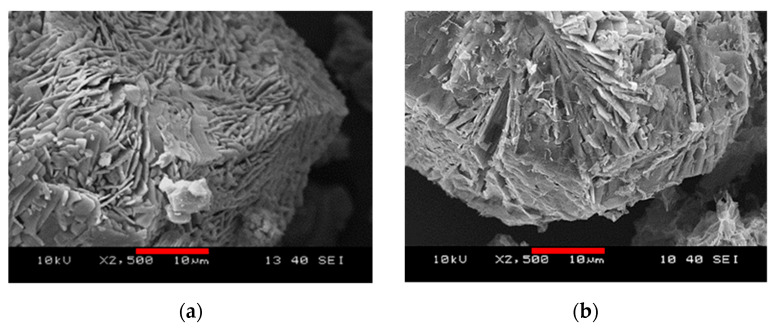
SEM images of (**a**) HKUST-1_110°C_85%RH and (**b**) HKUST-1@GO-2_110°C_85%RH. The scale bar is 10 µm.

Elevated baselines, peak widening and the appearance of new peaks indicate structural change in both sets of the activated samples, independently of the added GO and the storage conditions. Although the characteristic peaks of HKUST-1 are still recognisable in the diffractograms of HKUST-1_110_11 and HKUST-1@GO_110_11, their broadening and occasional changes in the intensity ratios are obvious signs of certain structural changes. This may also correspond to the partial loss of micropore structure indicated by the nitrogen adsorption isotherms. Samples exposed to high relative humidity after evacuation (label 110_85) display XRD patterns that are completely different. As a result of XRD phase analysis based on the powder XRD reference patterns in the PDF4+ (Release 2020) database, the starting HKUST-1 material and a degradation product, namely hydrogen triaqua benzene-1,3,5-tricarboxylate copper(II) [Cu(OOC)_2_(C_6_H_3_COOH)·3H_2_O, PDF_00-064-1336], were identified as minor components. However, the principal product(s) of the decomposition reaction could not be identified. The disintegration of the pore structure is also corroborated by the corresponding nitrogen adsorption isotherms and the thermal analysis (Figure 7f and Figure 8). The structural change is also clearly visible in SEM images of the vapor-treated samples (Figure 10). In the high humidity environment following the activation at 110 °C, the formerly polyhedral crystals become lamellar, even in the presence of GO.

## 4. Conclusions

While HKUST-1 is an outstanding candidate for the storage of green energy gases or CO_2_ capture, its sensitivity to humid conditions challenges such applications. In composite systems, this drawback may be diminished or eliminated. In this work, HKUST-1@GOs were synthesized under solvothermal conditions by adding graphene oxide (GO) suspensions to the precursor mixture of HKUST-1. GO affects the crystal growth. On the one hand, by increasing the pH, it influences the protonation of H_3_BTC and thus the HKUST-1 nucleation. On the other hand, it can physically inhibit the crystal growth and, at high concentration (2.5 g·L^−1^), lead to the formation of rod- and flower-like non-porous structures. At lower concentrations, GO had no significant influence on the adsorption of clean energy gases under atmospheric conditions. The measured adsorption capacities agree well with data published by other groups. To the best of our knowledge, no such systematic study has been performed where the adsorption of all these three gases is investigated on the same HKUST-1@GO systems. The composite containing 16 wt.% GO (HKUST-1@GO-2) showed the best adsorption performance both for green energy gases and for CO_2_. This sample was investigated to understand the effect of GO in the composite on the protection against water. Pristine HKUST-1 and HKUST-1@GO-2 were exposed to relative humidities of 11% and 85% for 21 days after air-drying or activation (110 °C, 24 h).

The structural change results in the partial (110_11 label) or total (110_85 label) collapse of the pore network observable from the N_2_ adsorption–desorption isotherms. It can be assumed that as a combined effect of drying and the level of relative humidity, the crystal structure of HKUST-1 transforms partly into Cu(OOC)_2_(C_6_H_3_COOH)·3H_2_O) but in majority into further crystalline component(s), presumably via varying degrees of protonation of the carboxylate groups. Low temperature nitrogen adsorption was a more sensitive indicator of the textural changes than XRD. The protective effect of GO was clearly observable by comparing the isotherms of air-dried HKUST-1 and HKUST-1@GO-2 after exposure to 85% RH. The decrease in the values of *S*_BET_, *V*_tot_ and *V*_micro_ for the composite was half that for pristine HKUST-1. It was found that GO is able to save the copper–carboxyl coordination bonds, probably by sacrificing the ester groups formed during the solvothermal synthesis between ethanol and the carboxyl groups on the GO sheets. The complete removal of the water molecules during the activation leaves behind reactive Cu sites and easily accessible MOF porosity. In this case, therefore, the protecting effect of the GO ester groups cannot prevail.

## Figures and Tables

**Table 1:** Composition of HKUST-1@GO samples and their characteristic data from low temperature nitrogen adsorption–desorption isotherms *.

Sample.	*m*_GO_	*m*_dry_	Yield of HKUST-1	GO in Composite	*S*_BET_	*V*_micro_	*V*_tot_	*V*_meso_
	mg		%	%	m^2^ g^−1^	cm^3^ g^−1^		
GO	-	-	-	-	20	0.01	0.07	0.06
HKUST-1	0	213	84	0	1500	0.55	0.62	0.07
HKUST-1@GO-1	20	215	77	9	1470	0.54	0.61	0.07
HKUST-1@GO-1.5	30	227	78	13	1500	0.56	0.63	0.07
HKUST-1@GO-2	40	249	82	16	1550	0.57	0.65	0.08
HKUST-1@GO-2.5	50	212	64	24	560	0.21	0.27	0.06

* *m*_GO_ is the mass of graphene oxide (GO) added to the reaction mixture; *m*_dry_ is the mass of the composite after evacuation at 110 °C; *S*_BET_: apparent surface area; *V*_micro_, *V*_tot_ and *V*_meso_ *= V*_tot_ − *V*_micro_: micropore, total and mesopore volume, respectively.

**Table 2:** Comparison of the adsorption capacities at atmospheric pressure for N_2_ and H_2_ at −196 °C, CH_4_ and CO_2_ at 0 °C.

Sample	Uptake			
	cm^3^·(STP)·g^−1^			
	N_2_	H_2_	CH_4_	CO_2_
HKUST-1	402	261	31	91
HKUST-1@GO-1	394	223	28	196
HKUST-1@GO-1.5	406	206	29	194
HKUST-1@GO-2	418	247	31	192
HKUST-1@GO-2.5	174	87	12	52

**Table 3:** Apparent surface area (*S*_BET_), micropore (*V*_micro_), total (*V*_tot_) and mesopore volume (*V*_meso_) before and after exposure to relative humidity *.

Sample	*S*_BET_	*V*_micro_	*V*_tot_	*V*_meso_
	m^2^·g^−1^	cm^3^·(STP)·g^−1^		
HKUST-1	1500	0.56	0.61	0.05
HKUST-1_20_11	1410	0.53	0.59	0.06
HKUST-1_20_85	880	0.34	0.39	0.05
HKUST-1_110_11	450	0.18	0.44	0.26
HKUST-1_110_85	25	0.01	0.04	0.03
HKUST-1@GO-2	1550	0.57	0.65	0.08
HKUST-1@GO-2_20_11	1460	0.56	0.62	0.06
HKUST-1@GO-2_20_85	1200	0.46	0.50	0.04
HKUST-1@GO-2_110_11	470	0.18	0.36	0.18
HKUST-1@GO-2_110_85	65	0.03	0.05	0.02

* S_BET_: apparent surface area; *V*_micro_, *V*_tot_ and *V*_meso_ *= V*_tot_ − *V*_micro_: micropore, total and mesopore volume, respectively.

## Supplementary Materials

The following are available online at https://www.mdpi.com/2079-4991/10/6/1182/s1, Figure S1: N2 adsorption-desorption isotherms (−196 °C) of (**a**) the pristine materials and the composites on the same graph (**b**) the pristine GO, (**c**) HKUST-1 and (**d**–**g**) the composite materials with different GO content. The adsorption and desorption branches are marked with full and empty symbols, respectively; Figure S2: Integral pore size distribution of pristine GO, HKUST-1 and the composite materials with different GO content determined by BJH model from the adsorption branch of N_2_ adsorption-desorption isotherms. The validity of the method is limited to the 2–50 nm range; Figure S3: Initial section of the CO_2_ isotherms measured at 0 °C. Full isotherms are shown in Figure 6c; Figure S4: TG and limited temperature range DTG curves of the parent materials and the composites: (**a**,**b**) HKUST-1@GO-1, (**c**,**d**) HKUST-1@GO-1.5, (**e**,**f**) HKUST-1@GO-2, (**g**,**h**) HKUST-1@GO-2.5. GO (black) and HKUST-1 (blue) was plotted on all diagrams. Figure S5: N_2_ adsorption-desorption isotherms (−196 °C) of HKUST-1 (**a**) before and (**b**–**e**) after exposure to relative humidity. Air dried (20 °C, no vacuum) HKUST-1 exposed to (**b**) RH 11% and (**c**) RH 85%. Activated (110 °C, vacuum) HKUST-1 exposed to (**c**) RH 11% and (**d**) RH 85%. Figure S6: Integral pore size distribution of HKUST-1 before and after exposure to various RH media, determined by BJH method from the adsorption branch of N_2_ isotherms. The validity of the method is limited to the 2–50 nm range; Figure S7: N_2_ adsorption-desorption isotherms (−196 °C) of HKUST-1@GO-2 (**a**) before and (**b**-**e**) after exposure to relative humidity. Air dried (20 °C, no vacuum) HKUST-1@GO-2 exposed to (**b**) RH 11% and (**c**) RH 85%. Activated (110 °C, vacuum) HKUST-1@GO-2 exposed to (**c**) RH 11% and (**d**) RH 85%; Figure S8: Integral pore size distribution of HKUST-1@GO-2 before and after exposure to various RH media, determined by BJH method from the adsorption branch of N_2_ isotherms. The validity of the method is limited to the 2–50 nm range; Figure S9: Comparison of powder X-ray diffractograms of as received HKUST-1 (blue) and HKUST-1@GO-2 (orange) and after exposure to relative humidity with the same prehistory: (**a**) 20_11, (**b**) 20_85, (**c**) 110_11 and (**d**) 110_85. Table S1: Comparison of the H_2_, CH_4_ and CO_2_ adsorption performance of the reported materials with literature data; Table S2: Quantitative data from DTG curves in the water loss region of HKUST-1; Table S3: Quantitative data from DTG curves in the water loss region of HKUST-1@GO-2.
