# Supplementary material for: Graphene Oxide Protected Copper Benzene-1,3,5-Tricarboxylate for Clean Energy Gas Adsorption
Source: Nanomaterials (Basel). 2020 Jun 17;10(6):1182. doi: 10.3390/nano10061182 (PMC7353370; doi:10.3390/nano10061182)
Supplement: Supplementary file 1 [file nanomaterials-10-01182-s001.pdf]

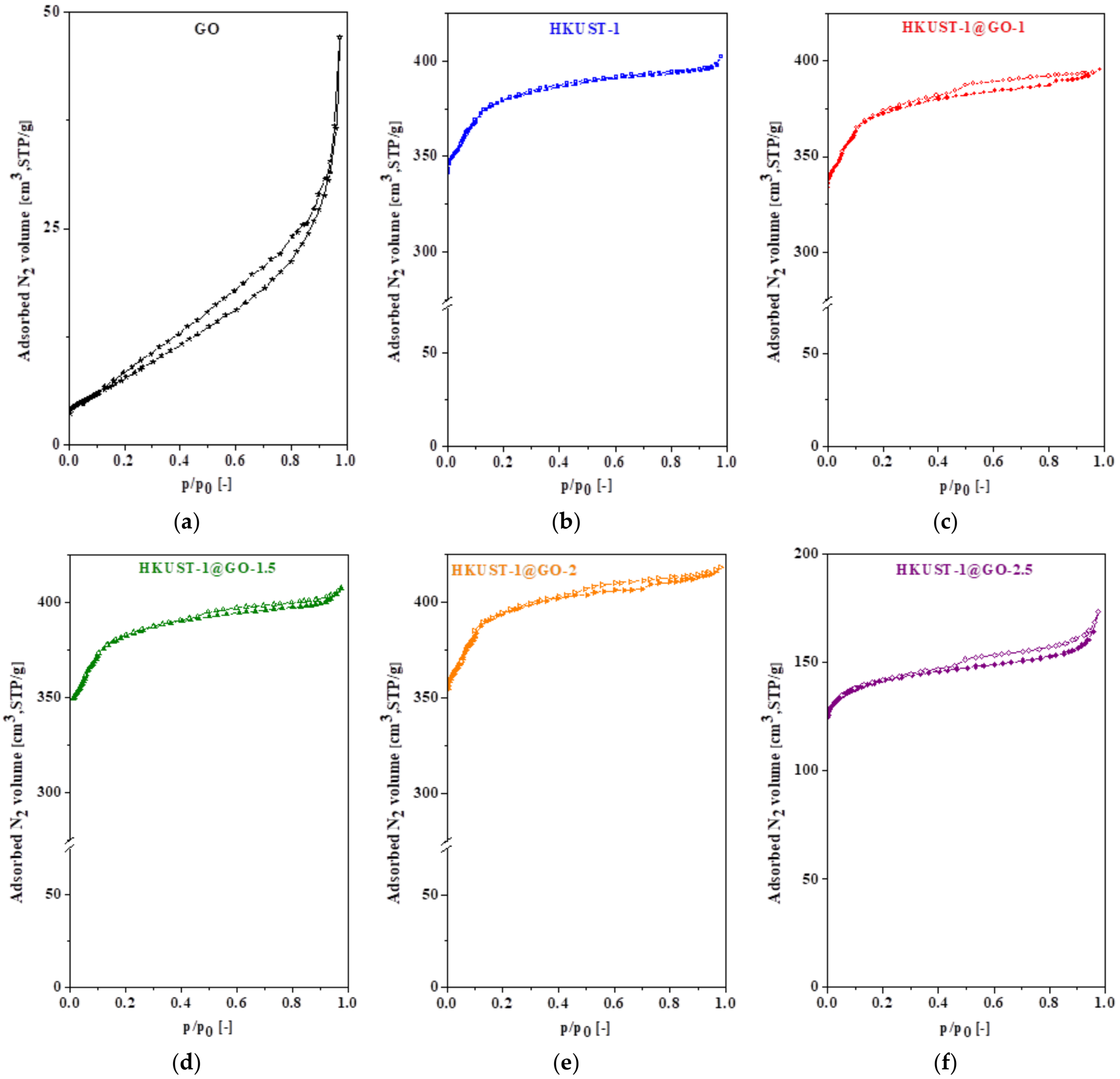

Figure S1. N_2 adsorption−desorption isotherms (−196 °C) of (**a**) the pristine GO, (**b**) HKUST-1 and (**c**–**f**) the composite materials with different GO content. The adsorption and desorption branches are marked with full and empty symbols, respectively.

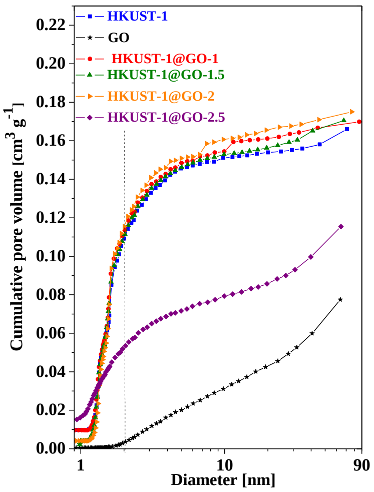

Figure S2. Integral pore size distribution of pristine GO, HKUST-1 and the composite materials with different GO content determined by BJH model from the adsorption branch of N_2 adsorption-desorption isotherms. The validity of the method is limited to the 2–50 nm range.

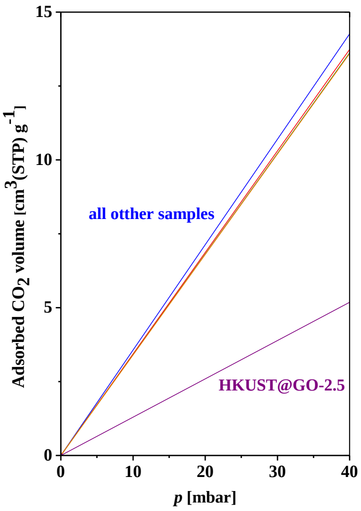

Figure S3. Initial section of the CO2 isotherms measured at 0 °C. Full isotherms are shown in Figure 6c.

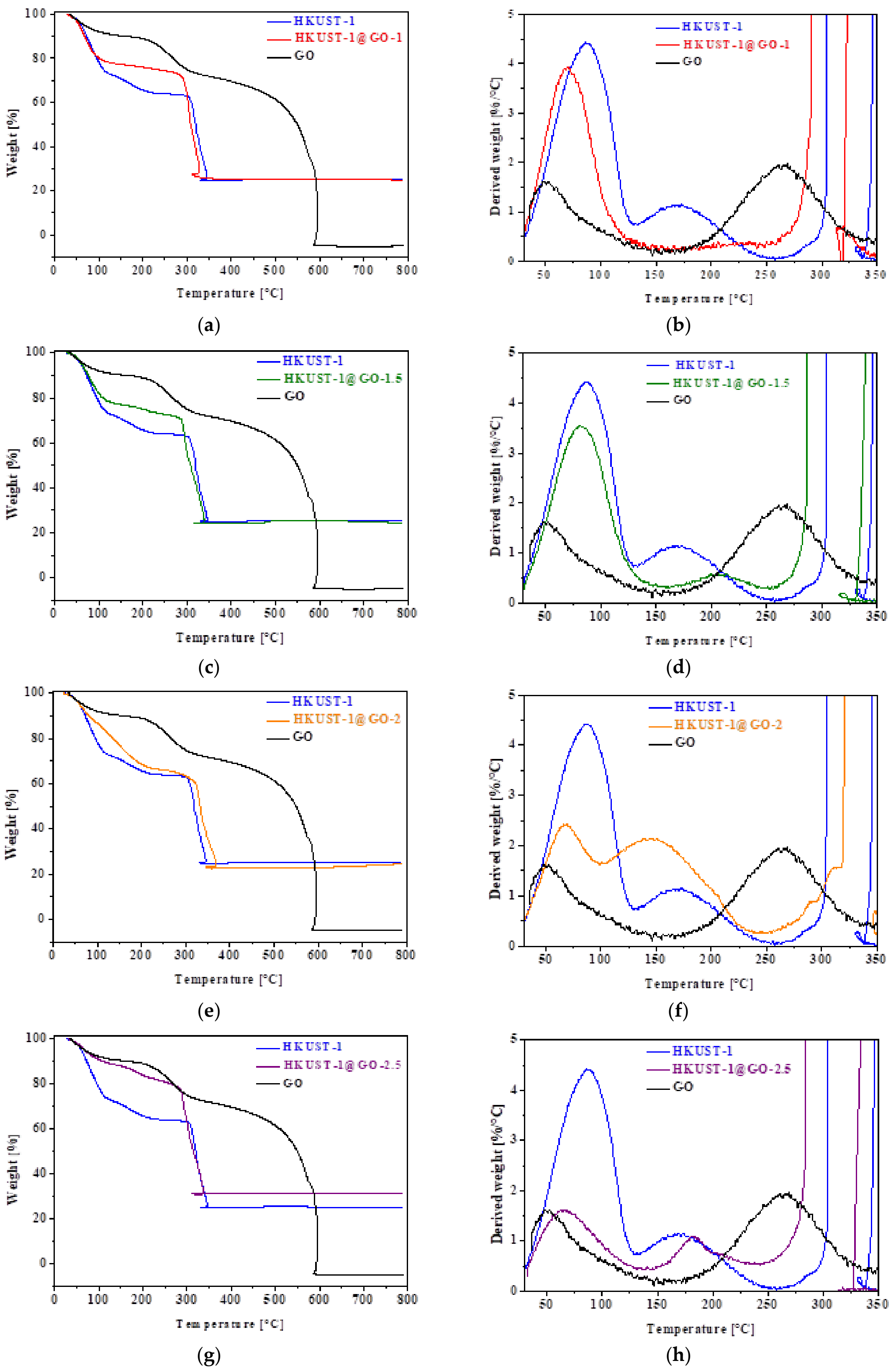

Figure S4. TG and limited temperature range DTG curves of the parent materials and the composites: (**a**,**b**) HKUST-1@GO-1, (**c**,**d**) HKUST-1@GO-1.5, (**e**,**f**) HKUST-1@GO-2, (**g**,**h**) HKUST-1@GO-2.5. GO (black) and HKUST-1 (blue) was plotted on all diagrams.

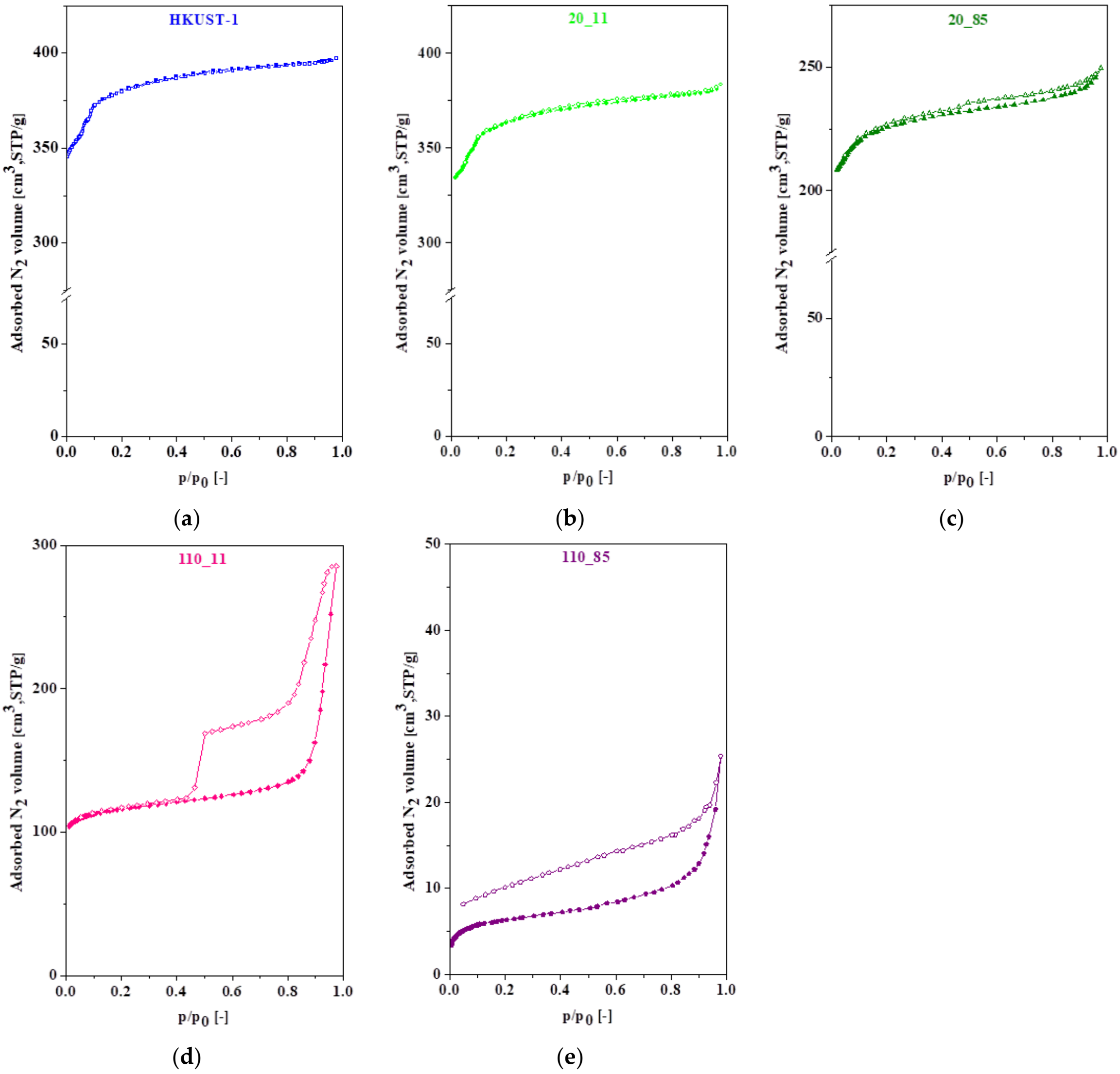

Figure S5. N_2 adsorption-desorption isotherms (−196 °C) of HKUST-1 (**a**) before and (**b**–**e**) after exposure to relative humidity. Air dried (20 °C, no vacuum) HKUST-1 exposed to (**b**) RH 11% and (**c**) RH 85%. Activated (110 °C, vacuum) HKUST-1 exposed to (**c**) RH 11% and (**d**) RH 85%.

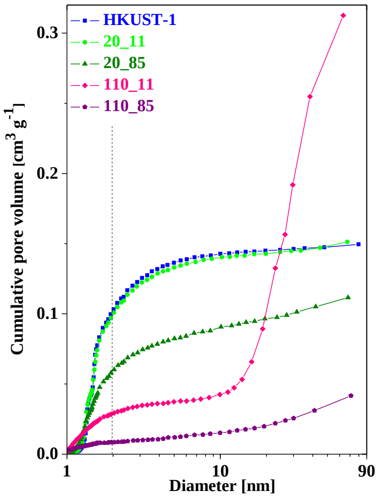

Figure S6. Integral pore size distribution of HKUST-1 before and after exposure to various RH media, determined by BJH method from the adsorption branch of N_2 isotherms. The validity of the method is limited to the 2–50 nm range.

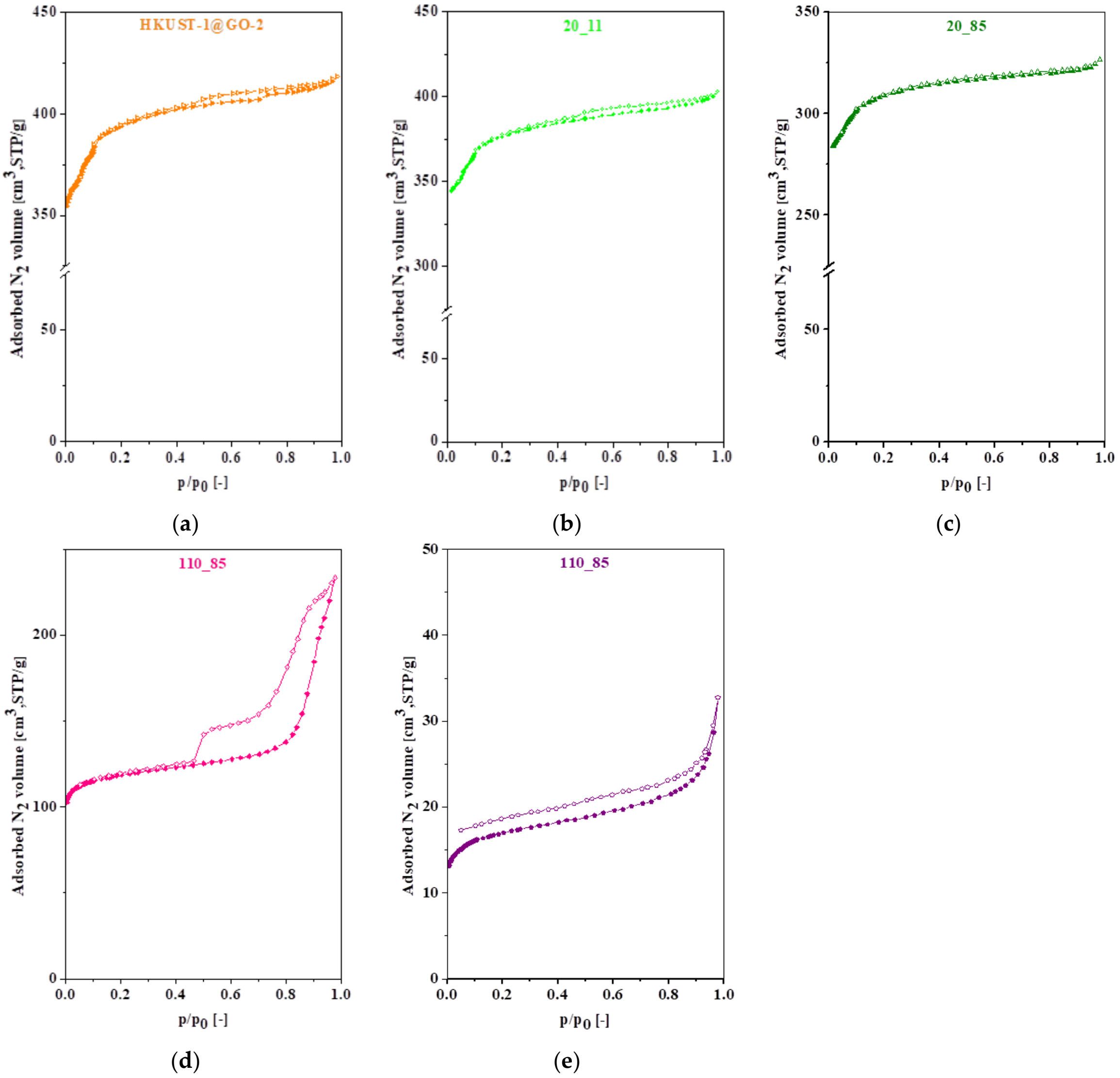

Figure S7. N_2 adsorption-desorption isotherms (−196 °C) of HKUST-1@GO-2 (**a**) before and (**b**–**e**) after exposure to relative humidity. Air dried (20 °C, no vacuum) HKUST-1@GO-2 exposed to (**b**) RH 11% and (**c**) RH 85%. Activated (110 °C, vacuum) HKUST-1@GO-2 exposed to (**c**) RH 11% and (**d**) RH 85%.

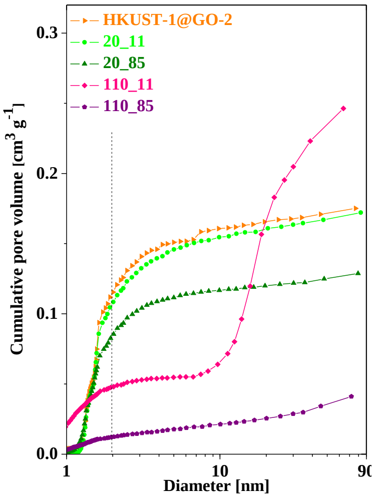

Figure S8. Integral pore size distribution of HKUST-1@GO-2 before and after exposure to various RH media, determined by BJH method from the adsorption branch of N_2 isotherms. The validity of the method is limited to the 2–50 nm range.

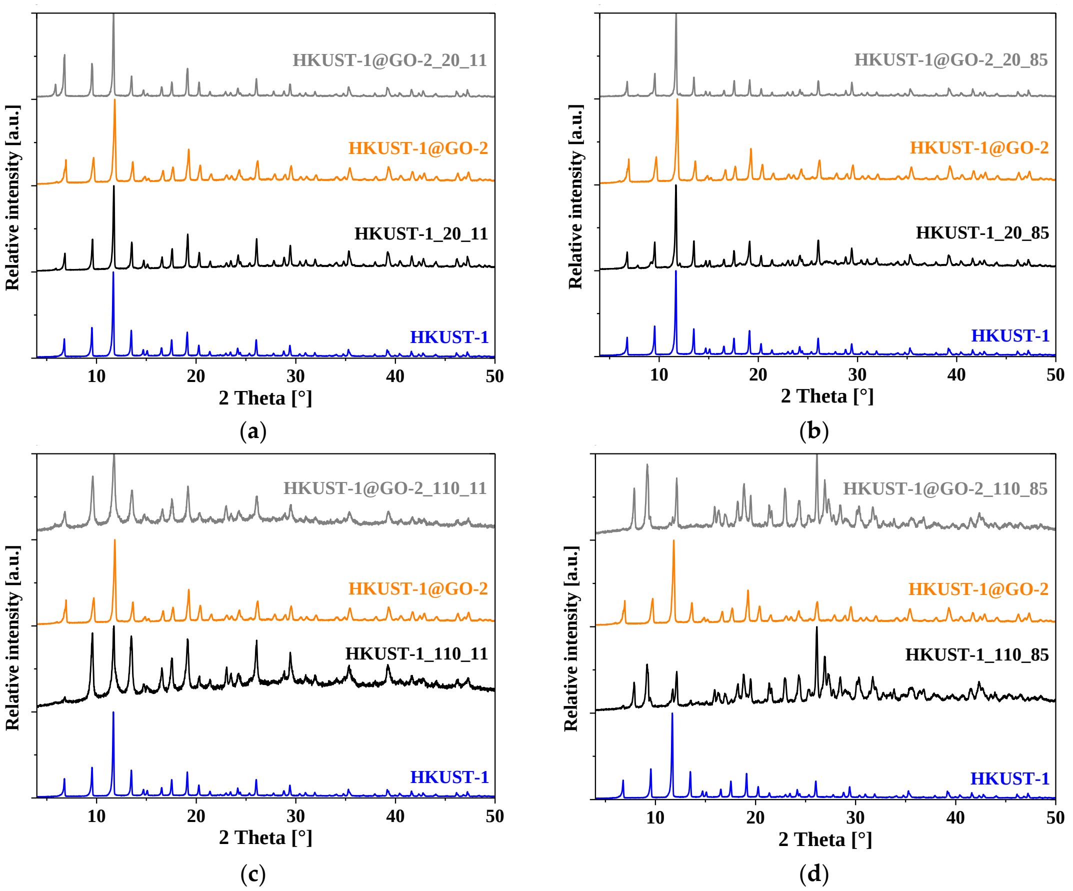

Figure S9. Comparison of powder X-ray diffractograms of as received HKUST-1 (blue) and HKUST-1@GO-2 (orange) and after exposure to relative humidity with the same prehistory: (**a**) 20_11, (**b**) 20_85, (**c**) 110_11 and (**d**) 110_85.

Table S1. Comparison of the H_2, CH_4 and CO_2 adsorption performance of the reported materials with literature data*.

Sample	GO in composite	S_{BET}	Adsorbate	Pressure	Temperature	Adsorption capacity	Ref.
	wt.%	m² g⁻¹		bar	°C	mmol g⁻¹	
HKUST-1		1500	H_2	1	−196	11.5	This work
HKUST-1@GO	16	1550	H_2	1	−196	10.9	This work
HKUST-1		909	H_2	1	−196	11.15	63
HKUST-1@GO	1	989	H_2	1	−196	11.8	63
HKUST-1	0	1305	H_2	42	−196	9.5**	55
HKUST-1@GO	9	1532	H_2	42	−196	10**	55
HKUST-1		1131	H_2	1.15	25	0.06**	64
HKUST-1@GO	5	955	H_2	1.15	25	0.04**	64
HKUST-1		1500	CH_4	1	0	1.4	This work
HKUST-1@GO	16	1550	CH_4	1	0	1.4	This work
HKUST-1		1382	CH_4	1	0	1,2**	52
HKUST-1@GO	1	1677	CH_4	1	0	1,3**	52
HKUST-1		1137	CH_4	5.8	25	2.78	54
HKUST-1@GO	10	1259	CH_4	5.8	25	2.56	54
HKUST-1		1137	CH_4	65	25	7.53	54

HKUST-1@GO	10	1259	CH_4	65	25	8.4	54
HKUST-1		1500	CO_2	1	0	4.0	This work
HKUST-1@GO	16	1550	CO_2	1	0	8.6	This work
HKUST-1		1305	CO_2	1	0	6.39	55
HKUST-1@GO	9	1532	CO_2	1	0	8.26	55
HKUST-1		1193	CO_2	1	0	6.85	56
HKUST-1@GO	2	1554	CO_2	1	0	9.02	56
HKUST-1		1382	CO_2	1	0	6.49	52
HKUST-1@GO	1	1677	CO_2	1	0	8.19	52
HKUST-1		1580	CO_2	1	0	8**	59
HKUST-1@GO	1	1772	CO_2	1	0	9**	59
HKUST-1		892	CO_2	1	4	3.86	61
HKUST-1@GO	10	1010	CO_2	1	4	5.00	61
HKUST-1		933	CO_2	1	25	2.77	62
HKUST-1@GO	6.06	837	CO_2	1	25	3.37	62
HKUST-1		no data	CO_2	1	25	2.3	60
HKUST-1@GO	10	no data	CO_2	1	25	3.2	60
HKUST-1		1048	CO_2	5.5	32	1.8**	53
HKUST-1@GO	10	1015	CO_2	5.5	32	2.5**	53

* Adsorption capacities are reported at the highest pressure applied except ** values where the capacity at 1 bar was estimated from the published isotherms.

Table S2. Quantitative data from DTG curves in the water loss region of HKUST-1.

	Mass loss wt.%			
Peak/Sample	I. (or Ia and Ib)	II. (or IIa and IIb)	III.	Residual Mass
**HKUST-1**	**27**	**9**	**39**	**25**
**20_11**	**21**	**7**	**44**	**28,5**
**20_85**	**33**	**1**	**41**	**25,6**
**110_11**	**17**	**2**	**50**	**32**
**110_85**	**10 7**	**4 2**	**47.5**	**29.5**

Table S3. Quantitative data from DTG curves in the water loss region of HKUST-1@GO-2.

	Mass loss wt.%			
Peak/Sample	I. (or Ia and Ib)	II. (or IIa and IIb)	III.	Residual Mass
**HKUST-1@GO-2**	**14**	**20**	**43**	**23**
**20_11**	**15**	**15**	**44.5**	**26**
**20_85**	**35**	**3**	**39**	**23**
**110_11**	**19**	**2**	**50**	**29**
**110_85**	**9 6**	**4 3**	**48.5**	**29.5**
